# The efficacy of topical calcineurin inhibitor therapy for childhood vitiligo: a systematic review and meta-analysis of randomized controlled trials and prospective cohort studies^؆^

**DOI:** 10.1016/j.abd.2025.501230

**Published:** 2025-11-01

**Authors:** Tiande Jiang, Wenting Wu

**Affiliations:** Department of Dermatology, Peking University Third Hospital, Beijing, China

**Keywords:** Calcineurin inhibitor, Child, Phototherapy, Tacrolimus, Vitiligo

## Abstract

**Background:**

Topical calcineurin inhibitors (TCIs) are commonly used in the treatment of pediatric vitiligo; however, limited studies have specifically examined their efficacy in this patient population.

**Objective:**

This study aims to evaluate the efficacy of TCI therapy in the treatment of childhood vitiligo.

**Methods:**

A comprehensive search was conducted in MEDLINE, EMBASE, and Web of Science to identify prospective cohort studies and randomized controlled trials (RCT) evaluating the efficacy of TCI therapy for childhood vitiligo. The primary outcome was treatment success, defined as ≥50% repigmentation. A meta-analysis was performed when appropriate. And the certainty of evidence was graded based on the GRADE (grading of recommendations assessment, development and evaluation) approach.

**Results:**

Nine studies involving a total of 636 patients were included in this review. Five studies were included in the meta-analysis, and 2 RCTs met eligibility criteria with medium-quality results. The efficacy of TCI therapy for childhood vitiligo was comparable to that of topical corticosteroid (TCS) therapy (Risk Ratio [RR = 0.70], 95% Confidence Interval [95% CI 0.39‒1.25]). Additionally, low-certainty evidence indicates that the combination of TCI therapy with phototherapy demonstrated superior results compared to phototherapy alone (RR = 1.32, 95% CI 1.01‒1.73).

**Study limitations:**

The findings are based on a limited number of studies and patients.

**Conclusion:**

TCI therapy for childhood vitiligo appears to be non-inferior to TCS therapy. Moreover, the combination of TCI and phototherapy may offer superior results compared to phototherapy alone; however, this conclusion should be interpreted with caution due to the low certainty of evidence as assessed by GRADE.

## Introduction

Vitiligo is a condition characterized by the loss of functional melanocytes, leading to depigmented skin macules and patches.^1^ It can manifest at any age, with the prevalence of childhood vitiligo ranging from 0.0% to 2.16%, while adult vitiligo prevalence ranges from 0.0% to 2.28%.^1^ Although vitiligo is generally considered an asymptomatic condition, a study indicates that nearly 30% of children with vitiligo experience itching and pain.^2^ Childhood vitiligo can significantly impact not only the quality of life of affected children but also the emotional well-being of their caregivers.^2^, ^3^ Vitiligo is a challenging dermatologic condition that imposes a substantial psychological burden on both patients and their families.

Despite the proven efficacy of topical corticosteroids (TCS) in childhood vitiligo, concerns regarding the side effects of corticosteroids are widespread.^4^, ^5^, ^6^ Tacrolimus ointment, a topical calcineurin inhibitor (TCI), has been shown to promote melanogenesis by modulating keratinocyte growth and stimulating melanocyte migration.^7^, ^8^ Unlike TCS, long-term use of TCIs is associated with minimal side effects in patients with atopic dermatitis.^9^ According to the present research, there are a few systematic reviews combined with meta-analysis of TCI treatment for childhood vitiligo. Given this, it is crucial to evaluate the efficacy of TCIs specifically for childhood vitiligo, in order to establish evidence-based treatment protocols tailored to this population.

## Methods

This review was performed according to the Preferred Reporting Items for Systematic Reviews and Meta-Analysis (PRISMA) statement.^10^ The authors have registered this systematic review in the PROSPERO database, and the registration ID is CRD42024629173.

### Search strategy

Two reviewers (T.J. and W.W.) independently searched databases, which included MEDLINE, EMBASE, and Web of Science from all databases to November 25, 2024. Randomized controlled trials (RCTs) and prospective cohort studies were identified by the search, which used these terms: “child*”, “pediatric,” “vitiligo”, “tacrolimus”, “pimecrolimus”, “calcineurin inhibitor”. Studies published online, in print, or in press were all included.

The search strategy is: 1) Pubmed: ("child"[Title/Abstract] OR "pediatric"[Title/Abstract]) AND "vitiligo"[MeSH Terms] AND ("tacrolimus"[Title/Abstract] OR "pimecrolimus"[Title/Abstract] OR "calcineurin inhibitors"[MeSH Terms]); 2) Embase: ('child'/exp OR 'child'/ti,ab OR 'pediatric'/exp OR 'pediatric'/ti,ab) ; AND 'vitiligo'/exp AND ('tacrolimus'/exp OR 'tacrolimus'/ti,ab OR 'pimecrolimus'/ti,ab OR 'calcineurin inhibitors'/exp); 3) Web of Science: TS = ("child*" OR "pediatric*") AND TS = ("vitiligo") AND TS = ("tacrolimus" OR "pimecrolimus" OR "calcineurin inhibitor")

### Study selection

Two independent reviewers screened the titles and abstracts. If there were insufficient information in abstracts, the two reviewers would examine full-texts and resolve any differences by discussion. The inclusion criteria: 1) Participants were children (under 18 years old); 2) At least one group treated with TCI; 3) Diagnosis of vitiligo (including but not limited to non-segmental vitiligo, segmental vitiligo, and undetermined/unclassified vitiligo). The exclusion criteria: 1) Duplicate publication; 2) Papers with no available full texts; 3) Non-English papers.

### Data extraction

Two reviewers independently performed data extraction. The template for extraction was built through the pilot extraction of three selected papers. The following data items included the last name of the first authors, country of studies, year of publications, number of participants/lesions, type of treatments, and duration of treatments. If studies were published by the same author, two reviewers would check whether the data overlapped or not.

### Risk of bias assessment

The quality of the clinical trials has been assessed by two independent reviewers. Two reviewers assessed the risk of biases using the Cochrane Collaboration’s quality assessment tool (ROB-2), which included the assessment of selection bias, performance bias, detection bias, attrition bias, reporting bias, and other biases.^11^ The risk of biases of prospective cohort studies was assessed by the Newcastle-Ottawa Scale (NOS) within the Selection, Comparability, and Outcome categories.^12^ Disagreements were resolved through the two reviewers’ discussions.

### Grading the certainty of evidence

The authors graded the certainty of evidence of relevant outcomes based on current GRADE guidance, which was divided into four categories: high, moderate, low, and very low.^13^ This method was developed to grade the overall certainty of evidence.

### Outcome measures

The primary outcome of interest was the extent of repigmentation, defined as 75% or greater. However, two studies used a threshold of 50% or greater repigmentation as the criterion for success.^14^, ^15^ Given the limited number of studies included in the meta-analyses, the authors chose 50% or greater repigmentation as the main outcome for this review.

### Statistical analysis

The authors used the Review Manager, Version 5.2, to perform the quantitative meta-analyses, using the Mantel-Haenszel ((M-H)) method and calculating the pooled Risk Ratio (RR) with the corresponding 95% Confidence Intervals (95% CI). A random-effects model would be used.

## Results

### Search results

**Fig. 1:**
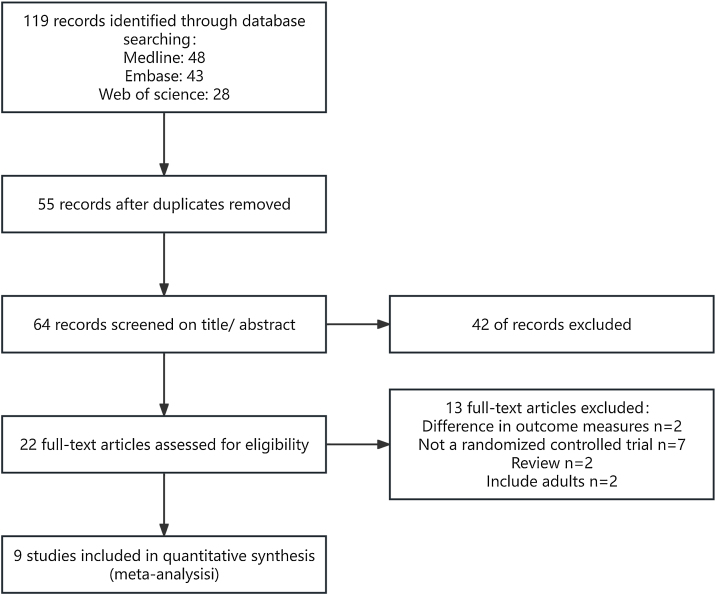
Flow diagram of the identification of selected studies.

A total of 9 articles were included in this review.^14^, ^15^, ^16^, ^17^, ^18^, ^19^, ^20^, ^21^, ^22^ The PRISMA flow diagram is presented in Fig. 1.

### Description of included studies

A total of 9 articles, comprising 636 patients, were included in this review (Table 1).^14^, ^15^, ^16^, ^17^, ^18^, ^19^, ^20^, ^21^, ^22^ Of these, 5 studies involving 323 patients were included in the meta-analysis.^15^, ^16^, ^17^, ^19^, ^20^ The age range of the study population was unspecified in 2 studies,^15^, ^22^ while the remaining studies included patients aged 2‒18 years. Four studies compared the efficacy of TCI with TCS,^14^, ^15^, ^17^, ^20^ three of which were included in the meta-analysis.^15^, ^17^, ^20^

**Table 1:** Characteristics of studies included in this review D, days; M, months; W, weeks.

Study			Sample Size		Intervention		Treatment duration
Author, year	Type	Country	Case	Control	Case	Control	
Alshiyab et al.,^21^ 2016	Randomized controlled trial	Jordan	25	25	0.1% Tacrolimus +308 nm excimer lamp	0.1% Tacrolimus	180 D
Alshiyab et al.,^22^ 2016	Randomized controlled trial	Jordan	25	24	0.1% Tacrolimus + pseudocatalase/superoxide dismutase gel	0.1% Tacrolimus	9 M
Dayal et al.,^16^ 2016	Open-label, prospective, clinical trial	India	20	20	0.03% Tacrolimus + NB-UVB	NB-UVB	24 W
Li et al.,^15^ 2018	Randomized controlled trial l	China	77/ 74	82/ 78	0.1% Tacrolimus +308 nm laser / 1% Pimecrolimus +308 nm laser	Halometasone +308 nm laser/ 308 nm laser	12 W
Ho et al., ^14^ 2011	Double-blind, randomized, placebo-controlled trial	Canada	31	30	0.1% Tacrolimus	0.05% Clobetasol propionate	24 W
Kőse et al.,^17^ 2010	Open label, single-center, comparative trial	Turkey	20	20	1% Pimecrolimus	0.1% Mometasone	12 W
Farajzadeh et al.,^18^ 2009	Single-blind, randomized placebo-controlled study	Iran	60	60	1% Pimecrolimus + microdermabrasion	1% Pimecrolimus	10 D
Yang et al.,^19^ 2009	Single blinded, randomized study	China	48	48	1% Pimecrolimus +308 nm laser	308 nm laser	15 W
Lepe et al.,^20^ 2003	Randomized double-blind trial	Mexico	20	20	0.1% Tacrolimus	0.05% Clobetasol propionate	8 W

Six studies examined the effects of TCI combined with other treatments compared to monotherapy.^15^, ^16^, ^18^, ^19^^,^^21^, ^22^ Among them, three studies compared TCI combined with phototherapy to phototherapy alone.^15^, ^16^, ^19^ Two of these studies specifically compared tacrolimus combined with phototherapy to phototherapy alone,^15^, ^16^ while the other two studies compared pimecrolimus combined with 308 nm excimer laser treatment to excimer laser alone.^15^, ^19^ Three studies were excluded from the meta-analysis.^18^, ^21^, ^22^

### Risk of bias assessment among included trials

**Fig. 2:**
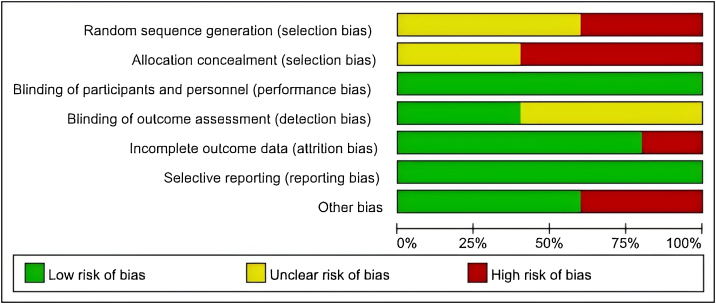
Risk-of-bias graph.

**Fig. 3:**
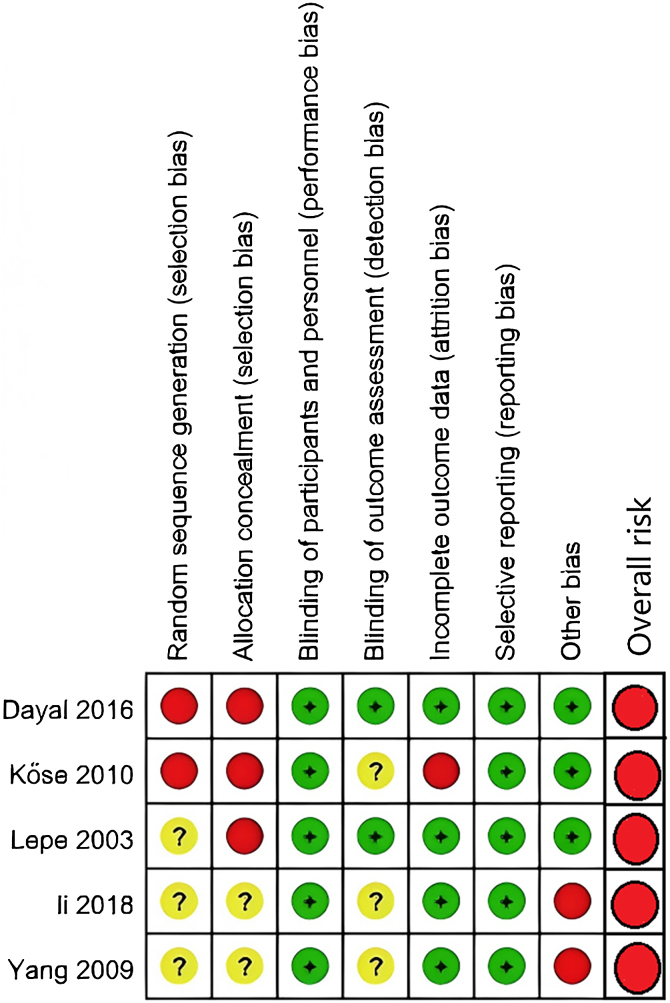
Risk-of-bias summary. Red ball, high risk; yellow ball, unclear risk of bias or some concerns; green ball, low risk.

Among the two prospective cohort studies, one received 4 points on the NOS,^17^ and the other received 6 points.^16^ According to the NOS criteria, studies scoring 7 or more points were considered high quality, while those scoring 3 or fewer points were classified as poor quality. Both studies were categorized as medium-quality studies. The results of the Randomized Controlled Trials (RCTs) are summarized in Fig. 2, Fig. 3. All the studies in Fig. 3 presented at least one domain with a high risk of bias, and this would lead to an overall risk of high risk.

### Effect of interventions

#### TCI versus TCS

**Fig. 4:**
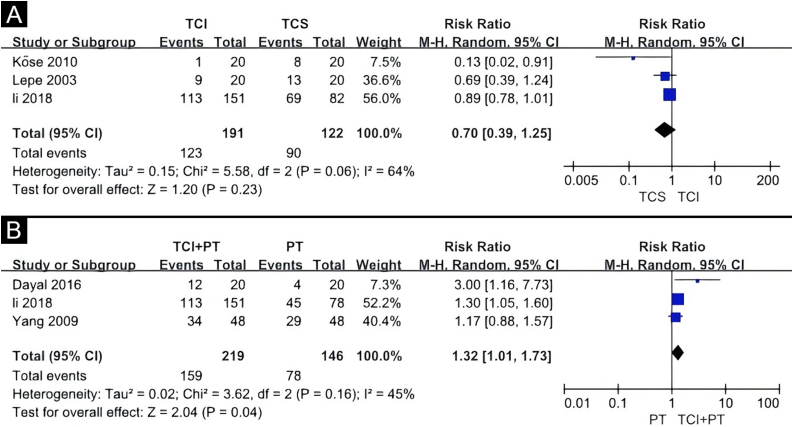
Forest plot meta-analysis of repigmentation rate of topical calcineurin inhibitor versus topical corticosteroid (A), topical calcineurin inhibitor and phototherapy combination therapy versus phototherapy alone (B). CI, Confidence Interval; TCI, Topical Calcineurin Inhibitor; TCS, Topical Corticosteroid; PT, Phototherapy.

The TCIs used in these studies included 0.1% tacrolimus and 1% pimecrolimus, while the TCS included halometasone, 0.05% clobetasol, and 0.1% mometasone. A random-effects model was applied, revealing no statistically significant difference between TCS and TCI in promoting 50% or more repigmentation (Risk Ratio [RR = 0.70], 95% Confidence Interval [95% CI 0.39‒1.25]) (Fig. 4A). Evidence for this was very low GRADE (Table 2). Another study not included in the meta-analysis also showed that TCS and TCI offered similar benefits.^14^

**Table 2:** Grading the certainty of evidence of topical calcineurin inhibitor versus topical corticosteroid.

Quality assessment							No of patients		Effect		Quality	Importance
No of studies	Design	Risk of bias	Inconsistency	Indirectness	Imprecision	Other considerations	TCI	TCS	Relative (95% CI)	Absolute		
>50% repigmentation												
3	Randomised Trials	Serious^a^	Serious^b^	No Serious Indirectness	Serious^c^	None	123/191 (64.4%)	90/122 (73.8%)	RR 0.7 (0.39 to 1.25)	221 fewer per 1000 (from 450 fewer to 184 more)	ÅOOO VERY LOW	
								65%		195 fewer per 1000 (from 396 fewer to 162 more)		

a Lack of information on randomization of outcome measure.

b I^2^ ＞ 50%.

c The population is small.

In a prospective randomized trial of treating with pimecrolimus, the pimecrolimus group was less effective for treating facial and neck lesions 64.5% (20/31) and more effective for acral lesions 80.0% (4/5).^15^ Conversely, the tacrolimus group showed lower effectiveness for acral lesions 50.0% (3/6), but higher effectiveness for trunk lesions 88.2% (30/34).^15^ In an open-label, single-center, comparative trial, pimecrolimus was less effective on the extremities and trunk compared to the face.^17^ A double-blind randomized trial study suggested that most of the repigmentation occurred on the face in patients treated with topical tacrolimus.^20^ Additionally, the study observed that TCS resulted in the development of a pigment island around the hair follicle, while the new pigmentation in the tacrolimus group showed a more uniform, centripetal pattern, mixing with the normal skin color.^20^ In a double-blind, randomized, placebo-controlled trial, TCS and TCI had similar effects on vitiligo in children, both in facial and non-facial areas.^14^ Regarding adverse events, a total of 60 patients were included. In the TCS group, 8.3% (5/60) of patients experienced skin atrophy, 6.7% (4/60) had telangiectasia, while 8.3% (5/60) of patients in the TCI group reported burning sensations or pruritus.^17^, ^20^

#### TCI combined with the other treatment versus monotherapy

Three quantitative meta-analyses were conducted. The first meta-analysis included three clinical trials comparing the efficacy of TCI combined with phototherapy versus phototherapy alone (Fig. 4B).^15^, ^16^, ^19^ The TCI treatment included 0.1% tacrolimus, 0.03% tacrolimus, and 1% pimecrolimus, while the phototherapy treatments included the 308 nm excimer laser and Narrowband Ultraviolet B (NBUVB). The combination of TCI and phototherapy showed a significantly superior effect on childhood vitiligo (RR = 1.32, 95% CI 1.01‒1.73) based on the random-effects model. Evidence for this was low GRADE (Table 3).

**Table 3:** Grading the certainty of evidence of topical calcineurin inhibitor and phototherapy combination therapy versus phototherapy alone.

Quality assessment							No of patients		Effect		Quality	Importance
No of studies	Design	Risk of bias	Inconsistency	Indirectness	Imprecision	Other considerations	Polytherapy	Monotherapy	Relative (95% CI)	Absolute		
>50% repigmentation												
3	Randomised trials	Serious^a^	No serious inconsistency	No serious indirectness	Serious^b^	None	159/219 (72.6%)	78/146 (53.4%)	RR 1.32 (1.01 to 1.73)	171 more per 1000 (from 5 more to 390 more)	ÅÅOO LOW	
								57.7%		185 more per 1000 (from 6 more to 421 more)		

a Lack of information on randomization of outcome measure.

b The population is small.

**Fig. 5:**
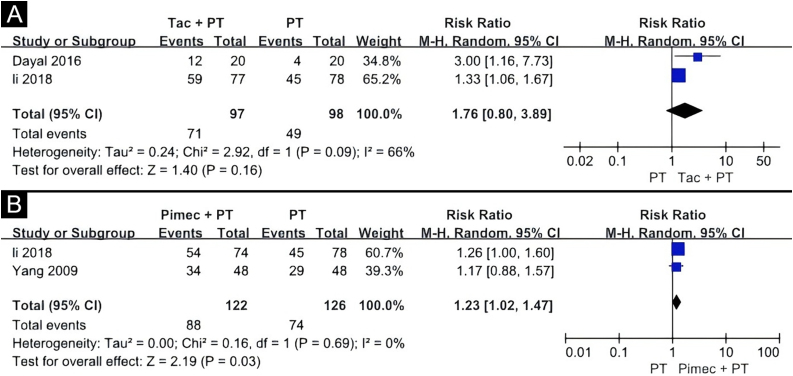
Tacrolimus and phototherapy combination therapy versus phototherapy alone (A), pimecrolimus and 308 nm excimer laser combination therapy versus 308 nm excimer laser alone (B). Tac, Tacrolimus; Pimec, Pimecrolimus.

The second meta-analysis included two clinical trials, comparing the efficacy of tacrolimus combined with phototherapy versus phototherapy alone (Fig. 5A).^15^, ^16^ One study used 0.03% tacrolimus with NBUVB, and the other used 0.1% tacrolimus with the 308 nm excimer laser. The results showed no statistical difference between the two groups (RR = 1.76, 95% CI 0.80‒3.89), according to the random-effects pooling of the results. Evidence for this was very low GRADE (Table 4). The response was found to be more effective on the face and trunk than on the extremities in an open-label study.^16^ Adverse effects reported in this study included erythema in 10.0% (2/20) patients, blistering in 5.0% (1/20) patients, and pruritus in 5.0% (1/20) patients.^16^

**Table 4:** Grading the certainty of evidence of tacrolimus and phototherapy combination therapy versus phototherapy alone.

Quality assessment							No of patien No of patients ts		Effect		Quality	Importance
No of studies	Design	Risk of bias	Inconsistency	Indirectness	Imprecision	Other considerations	Calci + exi	Exi	Relative (95% CI)	Absolute		
>50% repigmentation												
2	Randomised trials	Serious^a^	Serious^b^	No serious indirectness	Serious^c^	None	71/97 (73.2%)	49/98 (50%)	RR 1.76 (0.8 to 3.89)	380 more per 1000 (from 100 fewer to 1000 more)	ÅOOO VERY LOW	
								38.9%		296 more per 1000 (from 78 fewer to 1000 more)		

a Lack of information on randomization of outcome measure.

b I^2^＞.50%.

c The population is small.

The third meta-analysis involved two clinical trials comparing the combination of 1% pimecrolimus and 308 nm excimer laser treatment to 308 nm excimer laser alone for childhood vitiligo (Fig. 5B).^15^, ^19^ The random-effects pooling revealed that the combination therapy had a significantly superior effect (RR = 1.23, 95% CI 1.02‒1.47). Evidence for this was low GRADE (Table 5). In a right/left comparative, single-blinded trial, of the 48 patients receiving the 308 nm excimer laser, 16.7% (8/48) experienced mild burning sensations, 6.3% (3/48) had blisters, and 14.6 (7/48) had pruritus.^19^ Some of these patients experienced worsened symptoms after using pimecrolimus.^19^

**Table 5:** Grading the certainty of evidence of pimecrolimus and 308 nm excimer laser combination therapy versus 308 nm excimer laser alone.

Quality assessment							No of patients		Effect		Quality	Importance
No of studies	Design	Risk of bias	Inconsistency	Indirectness	Imprecision	Other considerations	Poly	Mono	Relative (95% CI)	Absolute		
>50% repigmentation												
2	Randomised trials	Serious^a^	No serious inconsistency	No serious indirectness	Serious^b^	None	88/122 (72.1%)	74/126 (58.7%)	RR 1.23 (1.02 to 1.47)	135 more per 1000 (from 12 more to 276 more)	ÅÅOO LOW	
								59.1%		136 more per 1000 (from 12 more to 278 more)		

a Lack of information on the randomization of outcome measure.

b The population is small.

Three studies were not included in the meta-analysis.^18^, ^21^, ^22^ In these studies, TCI combined with microdermabrasion or with a 308 nm excimer lamp showed greater effectiveness than TCI alone.^18^, ^21^ However, the combination of tacrolimus and topical pseudocatalase/superoxide dismutase gel did not appear to offer additional benefit.^22^

## Discussion

The systematic review provided the first child-specific evidence supporting two pivotal conclusions: 1) TCIs achieved comparable ≥ 50% repigmentation rates to potent TCS (RR = 0.70, 95% CI 0.39‒1.25) with superior safety; 2) Efficacy was influenced by the anatomic site. The comparable efficacy between TCIs and potent TCS contrasted with data from both pediatric and adult studies.^23^ This divergence likely stemmed from developmental differences in skin biology: children's thinner epidermis may enhance TCI penetration, potentially compensating for lower anti-inflammatory potency. Recent 2024 consensus treatment recommendations published in JAMA Dermatology identified TCIs applied twice daily as evidence-based first-line therapies for managing pediatric and adolescent vitiligo.^24^ According to the present systematic review, both TCS and TCI demonstrated similar efficacy in treating childhood vitiligo (RR = 0.70; 95% CI 0.39‒1.25). These findings supported the use of TCIs as first-line therapy for pediatric patients with vitiligo.

However, this review had several limitations. First, heterogeneity among the studies included in the meta-analysis remained a concern. Second, the sample sizes (both in terms of the number of patients and lesions), follow-up durations, and the number of studies were relatively small. Third, the review might be limited by the risk of bias in the studies.

Most studies included in the analysis used 0.1% tacrolimus. However, it was challenging to obtain coverage in the U.S., as many insurance companies limit coverage to 0.03% tacrolimus for patients under 16 years of age. The authors did not find any literature comparing the efficacy of 0.03% tacrolimus and topical corticosteroids in children with vitiligo. In a systematic review about atopic dermatitis, a comparison with a commonly used mild topical corticosteroid showed that 0.03% tacrolimus was more effective, while no significant differences were found between 0.03% tacrolimus and mid-potency corticosteroids.^25^ It was hoped that future clinical studies would compare 0.03% tacrolimus with topical corticosteroids for the treatment of vitiligo in children.

Several other findings were reported in this systematic review: 1) The combination of TCIs with phototherapy showed some improvement in outcomes compared to phototherapy alone; however, the statistical significance was marginal, and the clinical relevance of this difference should be carefully considered; 2) Similarly, the combination of topical pimecrolimus with a 308 nm excimer laser showed slightly better results compared to excimer laser therapy alone. Despite these findings, the clinical significance of these improvements remains uncertain.

Tacrolimus (0.03%) + NB-UVB showed variable efficacy with face lesions exhibiting a good to excellent response (100%), compared to patches on the limbs (75%).^16^ Lesions on the face and neck (73.08%) were more affected than those on the limbs (63.64%) by 0.1% tacrolimus + NB-UVB.^15^ The limited efficacy in acral regions necessitated additional therapies (e.g., prolonged phototherapy sessions or surgical interventions or to improve the permeability of drugs), indirectly increasing cumulative costs due to sequential treatment failures.

The action of TCIs (such as tacrolimus) in vitiligo as: 1) TCIs downregulate proinflammatory cytokines, such as tumor necrosis factor, and induce anti-inflammatory cytokines, such as interleukin IL-10; 2) Promote the migration and proliferation of melanocytes and melanoblasts; 3) Induce melanogenesis through increasing tyrosinase expression and dopa oxidase activity; 4) Reduce oxidative stress and improved antioxidant capacity.^26^, ^27^, ^28^

Initial treatment with TCIs might cause local irritation, erythema, burning sensations, and itching.^29^ The risk of these side effects might be heightened in combination therapy. There were concerns about whether TCIs could increase the risk of cancer.^30^ However, two studies did not find significant evidence linking TCI use with an increased risk of skin cancer or lymphoma.^31^, ^32^

While TCI formulations generally carried higher upfront costs compared to TCS, the observed lower rates of treatment-disruptive adverse events might result in long-term cost savings through improved adherence and reduced complication management. Regional pharmacoeconomic analyses would be needed to optimize formulary decisions.

## Conclusion

In conclusion, TCI therapy for childhood vitiligo appears to be non-inferior to TCS therapy. Moreover, the combination of TCI and phototherapy may offer superior results compared to phototherapy alone; however, this conclusion should be interpreted with caution due to the low certainty of evidence as assessed by GRADE. TCI, or its combination with other treatments, is a promising option for pediatric patients with vitiligo. However, further clinical trials with larger sample sizes and more rigorous methodologies are needed for a more comprehensive quantitative analysis.

## ORCID ID

Wenting Wu: 0009-0008-0993-2783

## CRediT authorship contribution statement

Tiande Jiang: Study design and conception, data collection, analysis and interpretation of data, statistical analysis, writing of the article, or critical review of important intellectual content, final approval of the final version of the manuscript.

Wengting Wu: Data collection, analysis and interpretation of data, and statistical analysis.

## Financial support

None declared.

## Research data availability

The entire dataset supporting the results of this study was published in this article.

## Declaration of competing interest

None declared.
